# An effective AKT inhibitor-PARP inhibitor combination therapy for recurrent ovarian cancer

**DOI:** 10.1007/s00280-022-04403-9

**Published:** 2022-04-13

**Authors:** Jing Xu, Yi Gao, Xiaotian Luan, Ke Li, Jing Wang, Yilin Dai, Mingyi Kang, Chong Lu, Minhua Zhang, Chris X. Lu, Yu Kang, Congjian Xu

**Affiliations:** 1 Obstetrics and Gynecology Hospital of Fudan University, Shanghai, 200011 China; 2 Shanghai Key Laboratory of Female Reproductive Endocrine Related Diseases, Shanghai, 200011 China; 3 Obstetrics and Gynecology, Shanghai Medical College, Fudan University, Shanghai, China; 4 Laekna Therapeutics Shanghai Co., Ltd., Shanghai, China

**Keywords:** Ovarian cancer, AKT inhibitor, PARP inhibitor, Combination therapy

## Abstract

**Background:**

Although the use of PARP inhibitor has received considerable amount of attention in ovarian cancer, PARP inhibitor resistance still emerges with disease progression. PI3K/AKT pathway inhibitors have been proposed to synergize with PARP inhibition to slow tumor growth, but the exact molecular mechanisms are still elusive.

**Methods:**

Utilizing tumor samples from recurrent EOC patients with platinum resistance and prior PARP inhibitor use, Mini PDX and PDX models were established to study the anti-tumor effect of AKT inhibitor (LAE003) and LAE003/PARP inhibitor (Olaparib) in combination. Five ovarian cancer cell lines were treated with Olaparib or LAE003 or in combination in vitro. Cell viability and apoptosis rate were measured after the treatments. Combination index by the Chou–Talalay was used to evaluate in vitro combination effect of Olaparib and LAE003. The protein expression level of PARP1 and PAR was measured by Western blot in cell lines and by immunohistochemistry in PDX tumor tissues.

**Results:**

Tumor cells from two out of five platinum-resistant ovarian cancer patients previously treated with PARP inhibitor were sensitive to AKT inhibition in Mini-PDX study. Inhibition of AKT further increased the response of tumor cells to Olaparib in a PDX model derived from a recurrent platinum-resistant ovarian cancer patient. Additive anti-proliferation effect of LAE003 and Olaparib was also observed in three ovarian cancer cell lines with high PARP1 protein level. Interestingly, mechanism study revealed that AKT inhibition decreased PARP enzyme activity as measured by PAR level and/or reduced PARP1 protein level in the tumor cell lines and PDX tumor tissues, which may explain the observed combined anti-tumor effect of LAE003 and Olaparib.

**Conclusion:**

Collectively, our results suggest that the combination of AKT inhibitor and PARP inhibitor could be a viable approach for clinical testing in recurrent ovarian cancer patients.

## Introduction

Epithelial Ovarian Cancer (EOC) is the leading cause of gynecologic cancer death worldwide [1, 2], which accounts for approximately 85–90% of all ovarian cancers [3, 4]. It is estimated that about 50–60% of EOC patients have homologous recombination deficiency (HRD) [5, 6]. The discovery that tumor cells with HRD are particularly sensitive to PARP inhibition has revolutionized the treatment landscape for EOC [7, 8]. So far, three PARP inhibitors (Olaparib [9], Rucaparib [10, 11] and Niraparib [12]) have been approved by US Food and Drug Administration (FDA) for the treatment of patients with recurrent EOC. Although platinum sensitive recurrent EOC patients with HRD initially respond to PARP inhibitors, these patients eventually develop acquired resistance to platinum and PARP inhibitor treatment and the disease progresses [13, 14]. Currently, there is no good treatment option for the patients with platinum resistant and/or PARP inhibitor-resistant recurrent EOC. A high unmet medical need exists for this segment of patients.

The key mechanism for acquired resistance to PARP inhibitor is the restoration of efficient homologous recombination (HR) DNA repair in tumor cells through secondary genetic alterations (e.g., secondary mutations in BRCA1/2 or other critical genes on the pathway of HR) [15, 16]. Therefore, combination of PARP inhibitor with drugs that suppress HR repair could be a rational approach to increase anti-tumor effect [17]. Inhibition of PI3K/AKT/mTOR pathway has been implicated to suppress HR repair by downregulating BRCA/RAD51 and increasing DNA damage [18, 19]. In a phase 1b clinical trial, combination treatment of Alpelisib (a PI3K inhibitor) and Olaparib (a PARP inhibitor) resulted in partial response in 10 out of 28 patients with recurrent EOC [20].

AKT is on the central node of PI3K/AKT/mTOR pathway and inhibition of AKT induces HRD in the tumor cells [21]. AKT is an AGC family kinase and there are three AKT isoforms (AKT1, AKT2, AKT3) [22, 23]. Several AKT inhibitors are currently under clinical development for solid tumors [24]. In the patients with platinum-resistant ovarian cancer, combination treatment of a pan-AKT inhibitor Afursertib, Carboplatin and Paclitaxel were efficacious with an object response rate (ORR) at 32.1% [25]. However, whether AKT inhibition could increase the response of recurrent EOC to PARP inhibitor has not been thoroughly evaluated.

In this study, we discovered an AKT inhibitor (LAE003/Uprosertib, a close analog of Afursertib) reduced the growth of tumor cells from platinum-resistant and PARP inhibitor-resistant ovarian cancer patients, and additively increased anti-tumor response when combined with PARP inhibitor Olaparib. In vitro mechanism study suggested that LAE003 may increase anti-tumor effect of Olaparib by further inhibiting PARP activity or downregulating PARP1 protein level. Our results warrant the clinical development of AKT inhibition/PARP inhibitor combination in advanced ovarian cancer patients.

## Methods

### Chemicals

LAE003 was provided by Laekna Therapeutics. Olaparib was purchased in Selleck (China, Catalog number: S1060).

### Patient sample collection

This study was approved by the ethics committee of Obstetrics and Gynecology Hospital of Fudan University (IRB number:2018–31). Five platinum-resistant EOC patients with prior PARP inhibitor treatment were recruited and four patients had confirmed resistance to PARP inhibitor. All patients signed informed consent. Tumor samples (biopsy sample from patient 3 and patient 4, surgical samples form patient 1 and patient 5, and ascites from patient 2) and paired whole blood samples were collected. Tumor samples were used for Mini PDX, PDX and IHC study.

### Mini-PDX in vivo study

Tumor samples were washed with buffer solution and non-tumor tissues/necrotic tumor tissues were removed in biosafety cabinet. The tumor tissues were cut into 1–3 mm^3^ fragments and digested with 1 × collagenase solution at 37 °C for 1–2 h. The resulting single cells were then collected with subsequent depletion of immune and stromal cells using microbeads. The tumor cell suspension was filled into Mini-PDX device and the devices were inoculated into both flanks of Balb/c nude mice (3 devices/mouse) for the Mini-PDX in vivo study. The next day, the inoculated mice were orally treated with either 30 mg/kg LAE003 (dissolved in 20% PEG400 and 1% DMSO) QD or vehicle (20% PEG400 and 1% DMSO) QD for 7 days. All mice were euthanized and the Mini-PDX devices were collected for CTG (Cell Titer-Glo) assay to determine the viability and cell proliferation of the tumor cells in the device.

### Patient-derived Xenograft mouse model

One portion of tumor biopsy sample from patient 3 was used to generated PDX model. In this study, P7 PDX was used for the evaluation of the anti-tumor effect of Olaparib and LAE003. The mice were orally dosed with 100 mg/kg Olaparib QD, 30 mg/kg LAE003 QD, 100 mg/kg Olaparib QD and 30 mg/kg LAE003 QD combo, or vehicle QD for 28 days. Tumor volume was monitored twice a week and body weight was measured daily. All mice were euthanized at the end of the study. Tumor weight was determined after collecting the tumors. These tumor samples were then generated as FFPE block for IHC study. Percentage of tumor growth inhibition (%TGI) was calculated with the formula [1 − (change of tumor volume in treatment group/change of tumor volume in control group)] × 100, which was used for the evaluation of anti-tumor efficacy.

### ctDNA NGS sequencing

Blood was collected from each patient for Next Generation Sequencing (NGS) of circulating tumor DNA (ctDNA) to identify of germline mutation and somatic mutation. Plasma was collected using Streck BCT and ctDNA was isolated using the QIAamp Circulating Nucleic Acid Kit (Qiagen, Cat# 55114) according to the manufacturer's protocol. The concentration of ctDNA was measured using the Qubit dsDNA HS Assay Kit (Thermo Fisher Scientific, Cat# Q32854) and quality was examined using the Agilent High Sensitivity DNA Kit (Cat# 5067-4626). ctDNA with yield greater than 5 ng without overly genomic DNA contamination was proceeded to library construction. Library construction was performed using KAPA HyperPlus Kit (KAPA Biosystems, Cat# KK8504) and target Enrichment was performed using Target Probes IGT Kit (iGene Tech, cat# T232 V2). NGS sequencing was performed on the NextSeq500 system (Illumina).

### Cell lines and culture condition

The OVCAR8, OVCA433, A2780, HEY, SKOV3 human ovarian cancer cell lines were obtained from American Type Culture Collection (ATCC, Manassas, VA, USA). All cell lines were maintained in RPMI 1640 medium supplemented with 10% fetal bovine serum (FBS, Gibco, USA) in 5% CO_2_ and 95% air at 37 °C.

### Growth viability assay and drug combination analysis

Cell counting kit-8 (Dojindo Molecular Technologies, Cat#CK04) assay was performed to measure the viability of ovarian cancer cell lines after Olaparib and LAE003 treatment. The 96-well plates were seeded with 1 × 10^3^ cells per well and treated with various concentrations of Olaparib for 96 h, or with LAE003 for 48 h, or with Olaparib and LAE003 combination for 48 h. The cell viability was measured according to the manufacture’s protocol. The half-maximal inhibitory concentration (IC_50_) values were calculated from dose–response curves. The combination index (CI) was calculated to judge the combination effect of Olaparib and LAE003 using CompuSyn software program [26]. At 50% of the effect, a CI value less than 1 suggested synergetic effect, and a CI greater than 1 suggested antagonistic effect (Fraction Affected = 0.5).

### Western blot analysis

Cells at 80% confluence were harvested after treated with LAE003 or Olaparib or in combination for different amount of time. The cells were lysed in RIPA buffer supplemented with 1% phenyl-methyl-sulfonyl-fluoride (PMSF). Cell lysates were collected by scraping and centrifuged at 12,000 rpm for 15 min. The concentration of protein samples was determined using BCA TM Protein Assay kit (Beyotime, Cat#P0010). Western blot was conducted by loading 30 μg of total protein from each sample onto 10% SDS-PAGE gel. The gel was transferred to a nitrocellulose membrane. The membrane was blocked with 5% nonfat milk in TBS-T for 1 h at room temperature and was incubated with primary antibodies at 4 °C overnight. Following primary antibodies were used: PARP1 (Cell Signaling Technology, Cat#9532, 89 kDa), PAR (EMD Millipore, Cat#MABC547, 160 kDa), GAPDH (Cell Signaling Technology, Cat#2118, 37 kDa). Finally, the membranes were incubated with HRP-linked anti-Rabbit (Cell Signaling Technology, 5127), or HRP-linked anti-Mouse secondary antibodies (Cell Signaling Technology, 43593) at 4 °C for 1.5 h. The signal was recorded using ImageQuant LAS 4000 Mini system.

### Apoptosis analysis

Apoptosis assay was carried out using Annexin V-FITC/PI staining (BD Biosciences, Cat#556547) with flow cytometry. Cells were washed with cold PBS for three times and resuspended in 1 × binding buffer after 48 h treatment with Olaparib and LAE003 at IC_50_ concentration (Table 3). Cells (1 × 10^5^) were then incubated with 5 µL annexin V-FITC and 5 µL propidium iodide (PI) for 15 min at ambient temperature (25 °C) in the dark. After the addition of 400 µL of 1 × binding buffer, the rate of cell apoptosis was measured using flow cytometer (Beckman Coulter).

### Immunohistochemistry analysis

One portion of tumor samples from the patients or PDX samples were fixed in 10% neutral formalin for 24 h to generate FFPE block. For ascite sample, tumor cells were collected by centrifugation. Cell pellet was fixed and used for FFEP block generation. Antigen retrieval was performed using citrate buffer (0.1 mol/L, pH 6.0) with a steamer. 4 µm sections were cut and mounted onto the slides. The slides were incubated with primary antibodies (anti-PARP1, Cell Signaling Technology, Cat#9532, 1:200 dilution; anti-PAR, EMD Millipore, Cat#MABC547, 1:200 dilution; anti-PTEN, Cell Signaling Technology, Cat#9559, 1:400 dilution; anti-pS473 AKT, Cell Signaling Technology, Cat#4060, 1:400 dilution in blocking solution) overnight at 4 °C. Slides without primary antibody incubation were used as negative control. Goat anti-rabbit horseradish peroxidase–conjugated secondary antibody or Goat anti-mouse horseradish peroxidase–conjugated secondary antibody was used. The chromogenic reaction was performed with DAB. For PTEN IHC and pAKT IHC, staining intensity and area was scored as 0–3 scale and the final score was given by multiplying staining intensity score and staining area score. The image was graded as negative (−) if the final score was less than 1. The image was graded as + for score between 1 to 3, +  + for score between 4 and 6 and +  +  + for score between 7 and 9. The score for each sample was given by two independent pathologists. For PARP1 IHC and PAR IHC, the number of tumor cells with positive IHC staining was counted in 5 random fields of each section at × 400 magnification. Staining level was given by multiplying staining intensity score and staining area score.

## Results

### AKT inhibition slowed the growth of tumor cells from platinum-resistant recurrent EOC patients

To understand whether the tumor cells with acquired resistance are sensitive to AKT inhibition, Mini-PDX platform was used. Five platinum-resistant EOC with prior PARP inhibitor treatment were recruited. Among these patients, four had confirmed resistance to PARP inhibitor. Table 1 summarized the basic clinical characteristics of the patients. Genetic alteration in the tumor were also analyzed by blood ctDNA NGS sequencing. As it has been reported that tumor cells with PI3K/AKT/mTOR pathway activation are likely to be more sensitive to AKT inhibition [27], baseline PI3K/AKT/mTOR pathway activation status as assessed by PTEN IHC and pAKT IHC was evaluated. Table 2 summarized the information of genetic alteration, PTEN IHC, pAKT IHC, and PARP1 IHC status of the tumor samples.

**Table 1:** Baseline patient characteristics for Mini-PDX study

Characteristics	No. (%)
Age in years at the time specimen collection, median (range)	56.5 (50–63)
Clinical TNM staging at ID, no	
T1	0 (0)
T2	0 (0)
T3	2 (40)
T4	1 (20)
T-unknown	2 (40)
N0	0 (0)
N1	1 (20)
N-unknown	4 (80)
M0	2 (40)
M1	0 (0)
M-unknown	3 (60)
Prior PARP inhibitor use	5 (100)
Resistance to PARP inhibitor	4 (80)
Resistance to PARP inhibitor—unknown	1 (20)
Prior platinum use	
Resistance to platinum	5 (100)

**Table 2:** Mini-PDX results and biomarker status of five EOC patients

Patient ID	1	2	3	4	5
Cell viability after AKTi treatment in Mini PDX (% vehicle, Mean ± SEM)	134.16 ± 7.05	N/A	47.26 ± 3.84^a^	128.08 ± 15.62	39.78 ± 9.67^a^
Genetic alteration (blood ctDNA)	MSH2 R929Q BRCA1 N704Cfs*7	MSH6 P1082S BRCA1 P1099Lfs*10 NTRK1 D109G TERT V251I RICTOR T1198A CHEK2 D488V	TSC1 F1059L BRCA2 I1418M RAD51D K91fs CSMD3 E795V TP53 N239S	MET V1070M TP53 C242Afs*5	BRCA1 R71T FOXM1 M727V
PTEN IHC	** + + **	** + **	** + **	** + **	** + + **
pAKT IHC	**–**	**–**	**–**	** + **	**–**
PARP1 IHC score	**0.28**	**0.33**	**0.25**	**0**	**0**

^a^*p* < 0.05 comparing to vehicle treated group (unpaired Student’s *t* test)

IHC score is indicated in bold.

**Fig. 1:**
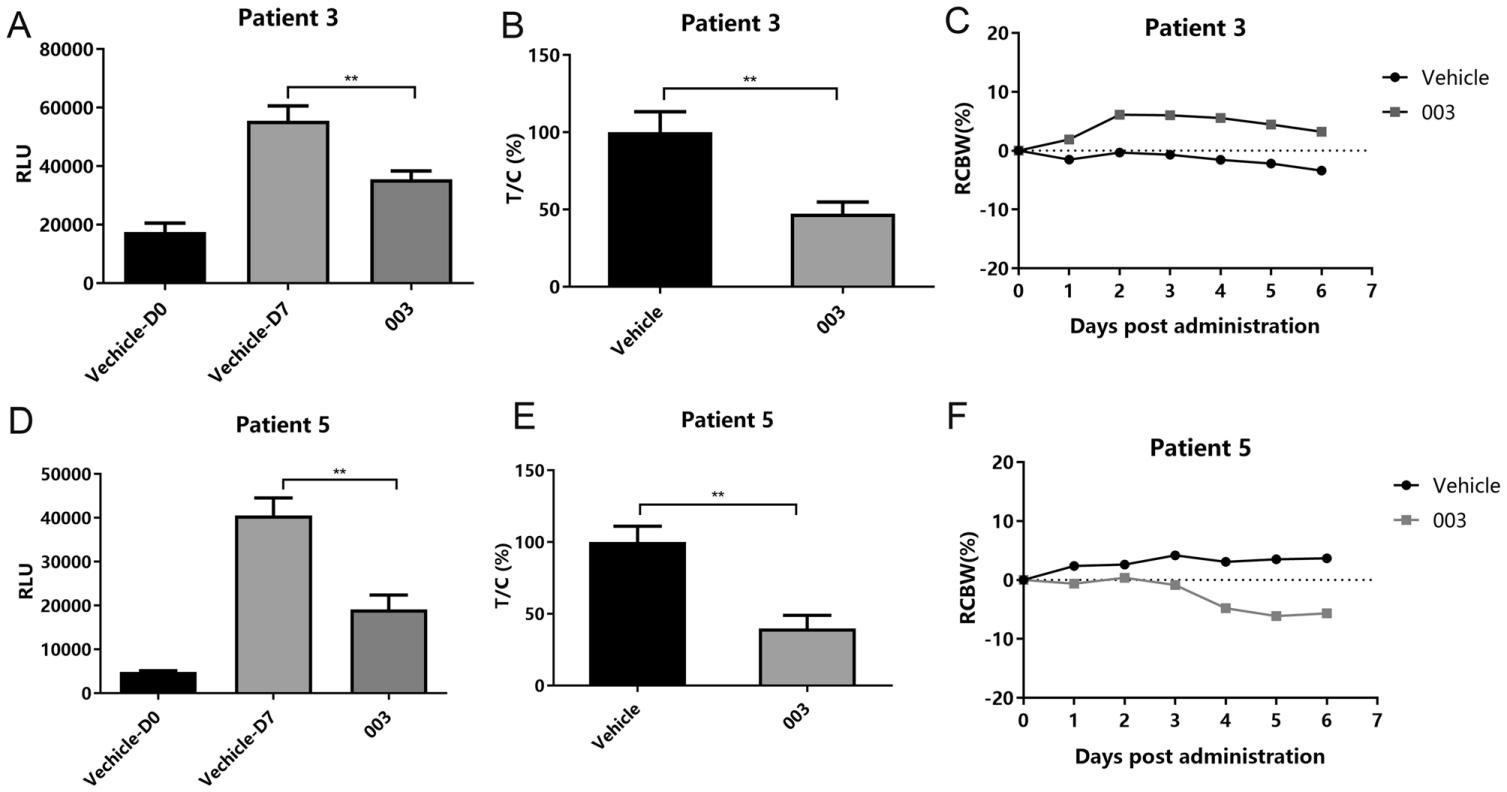
Tumor cells from Mini-PDX study of patient 3 and patient 5 were sensitive to LAE003 treatment. **A** Relative luciferase units (RLUs) of tumor cells measured by Cell Titer-Glo assay in Mini-PDX study of patient 3. **B** Relative viability of tumor cells in Mini-PDX study of patient 3 after normalizing to vehicle control group. **C** Body weight change of Mini-PDX study of patient 3. **D** RLUs of tumor cells measured by Cell Titer-Glo assay in Mini-PDX study of patient 5. **E** Relative viability of tumor cells in Mini-PDX study of patient 5 after normalizing to vehicle control group. **F** Body weight change of Mini-PDX study of patient 5. Data was presented as Mean ± SEM (*n* = 6/group). **P < 0.01, Student’s *t* test

Mini-PDX study was conducted using tumor biopsy or surgical tissue samples from these patients. Tumor cells were isolated from these samples and loaded into Mini-PDX devices. The mice inoculated with devices containing tumor cells were orally given an AKT inhibitor (LAE003/Uprosertib, a close analog to Afursertib) at 30 mg/kg QD or vehicle control for 7 days. At the end of the experiment, the mice were euthanized and cell viability of the tumor cells in the device was measured. As presented in Table 2 and Fig. 1, LAE003 treatment significantly reduced cell viability of the tumor cells from Patient 3 and Patient 5. The tumor cells from Patient 1 and Patient 4 were insensitive to LAE003 treatment. Result for Patient 2 was inconclusive, as the tumor cells did not grow in both vehicle and LAE003 treatment group during the course of Mini-PDX study.

### Combination of AKT inhibition and PARP inhibition had additive anti-tumor effect in a recurrent EOC PDX model

**Fig. 2:**
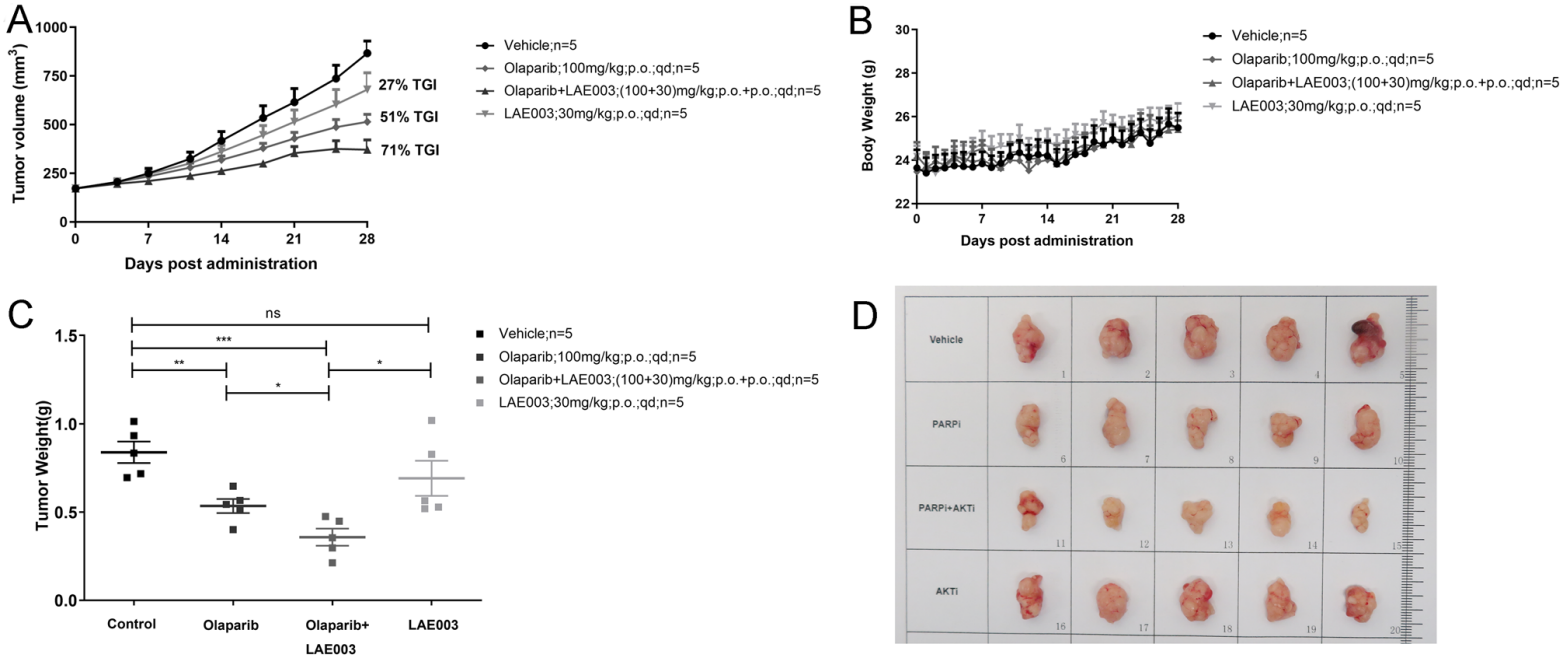
Combination of LAE003 and Olaparib had additive effect on slowing the tumor growth in PDX model from patient 3. **A** Tumor volume change after LAE003, Olaparib or LAE003/Olaparib combo treatment. **B** Body weight change after LAE003, Olaparib or LAE003/Olaparib combo treatment. **C** Tumor weight of LAE003, Olaparib or LAE003/Olaparib combo treatment groups at the end of the experiment. **D** Images of tumor samples collected at the end of the experiment. Data was presented as Mean ± SEM (*n* = 5/group). **P < 0.01, ***p < 0.001, Student’s *t* test

Based on the results of Mini-PDX study, a PDX model was further established using the tumor sample from patient 3, who had confirmed resistance to platinum and PARP inhibitor. In vivo efficacy study was carried out in the PDX model to evaluate whether inhibition of AKT could improve the response of tumor cells to PARP inhibitor. As showed in Fig. 2A, C and D, LAE003 single treatment mildly but significantly reduced tumor growth (27% TGI). Surprisingly, Olaparib single treatment was also effective (51% TGI), although the PDX model was derived from a patient with PARP inhibitor resistance. Combination of LAE003 and Olaparib further slowed the tumor growth (71% TGI) comparing to the single treatments. An additive anti-tumor effect of the combination was observed in vivo. Both LAE003 and Olaparib treatment did not affect body weight significantly (Fig. 2B).

### AKT inhibitor and PARP inhibitor additively reduced cell viability in ovarian cancer cell lines with high PARP1 protein expression level

To further evaluate the combination effect of PARP inhibitor and AKT inhibitor, five ovarian cancer cell lines (OVCA433, OVCAR8, A2780, SKOV3, and HEY) were studied. HRD status and PI3K/AKT/mTOR pathway genetic alteration of these five cell lines were checked in the database. As shown in Table 3, OVCA433, OVCAR8 and HEY cells had HR deficiency. All five cell lines bear certain genetic alterations of the genes in PI3K/AKT/mTOR pathway.

**Table 3:** The effect of LAE003, Olaparib and LAE003/Olaparib on ovarian cancer cell lines [28]

Cell line		OVCAR8	OVCA433	A2780	SKOV3	HEY
HRD status		Positive	Positive	Negative	Negative	Positive
PI3K/AKT/mTOR pathway mutation status [29]		AKT3 gain	AKT2 Start_Out Of Frame^[a]^	AKT3 gain PIK3CA mut (ms) PTEN mut (del)	PIK3CA mut (ms) PTEN hetloss	PIK3CA gain
IC_50_ (µM)	LAE003	7.975	1.913	2.852	6.347	5.915
	Olaparib	24.76	7.549	4.539	3.411	9.153
Combination Index (CI)		0.53268	0.49520	0.53024	1.6888	1.82563
Apoptotic Rate (%)	LAE003	5.07	5.409	2.47	8.06	5.75
	Olaparib	5.74	4.02	3.23	11.82	3.22
	LAE003 + Olaparib	11.18	10.059	7.41	10.27	4.54
Baseline PARP1 Level		High	High	High	Low	Low

[a]: CCLE data

**Fig. 3:**
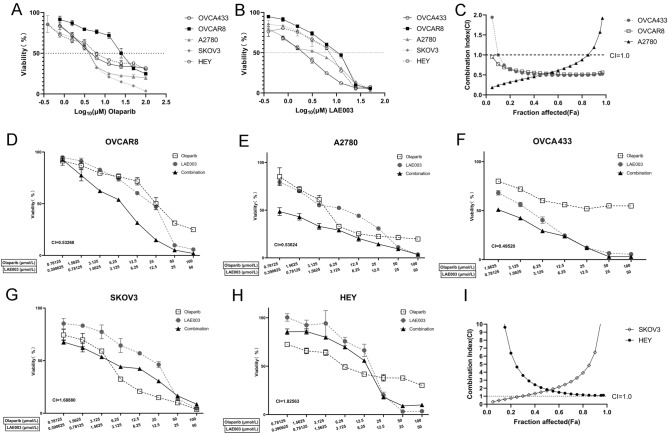
LAE003, Olaparib or LAE003/Olaparib combination reduced cell viability in ovarian cancer cell lines. **A** Olaparib dose dependently reduced cell viability in OVCA433, A2780, HEY, SKOV3 and OVCAR8 cell lines. **B** LAE003 dose dependently reduced cell viability in OVCA433, A2780, HEY, SKOV3 and OVCAR8 cell lines. **C** The additive effect of LAE003 and Olaparib combination treatment as analyzed using CI equation in OVCA433, A2780 and OVCAR8 cell lines. **D**–**H** LAE003, Olaparib or LAE003/Olaparib combination dose dependently reduced cell viability of OVCAR8, A2780, OVCA433, SKOV3, or HEY cell lines. **I** No additive effect of LAE003 and Olaparib combination treatment as analyzed using CI equation in SKOV3 and HEY cell lines. Data was presented as Mean ± SEM (triplicate/data point)

The five cell lines were treated with a range of concentrations of LAE003 or Olaparib. As shown in Fig. 3A and B, LAE003 and Olaparib dose dependently reduced cell viability in all the cell lines tested with varied IC_50_ values. Among these cell lines, OVCAR8 was the least sensitive to Olaparib and LAE003 single treatment (Table 3).

LAE003/Olaparib combination treatment was also carried out in these cell lines. Additive effect of the combination treatment on cell viability was only observed in three cell lines (OVCA433, OVCAR8 and A2780) (Fig. 3D–F) when comparing to the single treatments. Combining LAE003 and Olaparib did not further reduce cell viability in SKOV3 and HEY cells (Fig. 3G, H). Combination index (CI) was further calculated to assess the combination effect of Olaparib and LAE003 in these ovarian cancer cell lines. The CI values in OVCA433, OVCAR8 and A2780 cells were lower than 1.0 (Fig. 3C, Table 3), suggesting an additive effect of the two treatments. The CI values in SKOV3 and HEY cells were higher than 1, suggesting no combination effect (Fig. 3I, Table 3).

The observation that only a portion of the cell lines responded to LAE003 and Olaparib combination treatment was intriguing. The efficacy of combination treatment did not correlate with HRD status or PI3K/AKT/mTOR pathway genetic alteration. As previously reported [30], PARP1 expression level could drive anti-tumor activity of combination treatment with PARP inhibitor, PARP1 protein level was further studied in these ovarian cancer cell lines. As shown in Fig. 5A, high PARP1 protein level was observed in OVCA433, OVCAR8 and A2780 cells, while very low level of PARP1 protein was detected in SKOV3 and HEY cells. Interestingly, only the cell lines which had high expression level of PARP1 protein (OVCA433, OVCAR8, A2780) responded to the combination treatment (Fig. 3D–F). This suggested that PARP1 protein level in the tumor cells could be a potential marker to predict the combo effect, although more studies need to be carried out to further confirm this finding.

### Combination of AKT and PARP inhibitor increased cell apoptosis in ovarian cancer cell lines with high PARP1 protein expression level

**Fig. 4:**
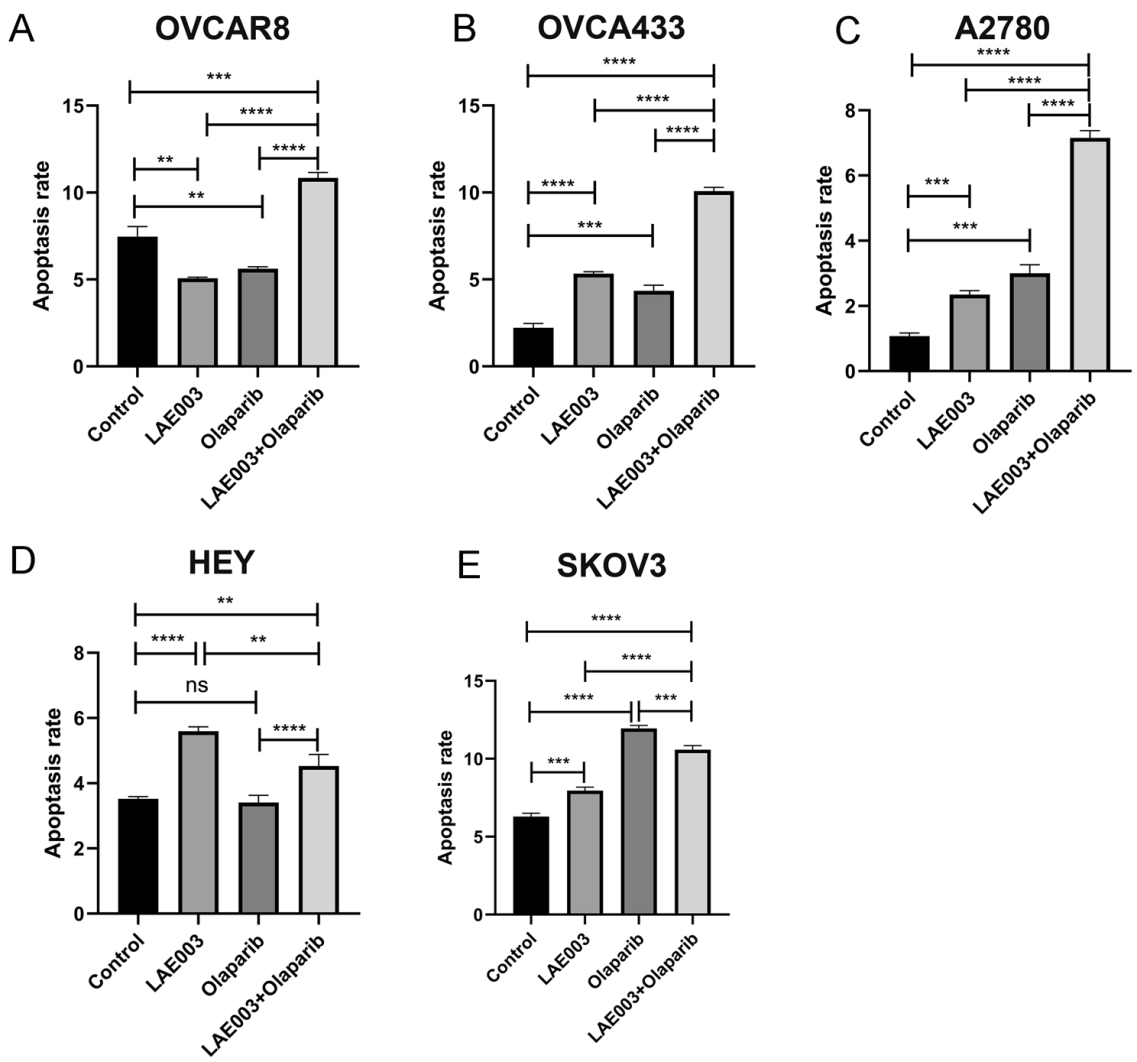
LAE003, Olaparib and LAE003/Olaparib combination treatment induced apoptosis in ovarian cancer cell lines. All cells were treated with compounds at IC_50_ concentrations determined from cell viability study. **A** OVCAR8 (8 µM LAE003; 25 µM Olaparib). **B** OVCA433 (2 µM LAE003; 8 µM Olaparib). **C** A2780 (4 µM LAE003; 5 µM Olaparib). **D** HEY (8 µM LAE003; 10 µM Olaparib). **E** SKOV3 (8 µM LAE003; 5 µM Olaparib). All cell lines were treated for 48 h. The percentage of apoptotic cells was determined by FACS. Data was presented as Mean ± SD (three independent experiments). ****P < 0.0001, ***P < 0.001, **P < 0.01 by Student’s *t* test

In addition to cell viability, apoptosis rate of these cell lines upon LAE003, Olaparib, or LAE003/Olaparib combination treatment was analyzed and cell lines without compound treatment were used as the control. All the cells were treated with LAE003 and/or Olaparib at IC_50_ concentrations determined from cell viability study. Comparing to single agent treatment, the combination treatment further increased the apoptosis rate in OVCA433, OVCAR8 and A2780 cells (Fig. 4A–C), but not in SKOV3 and HEY cells (Fig. 4D–E), consistent with the results from cell viability study. Taken together, these results demonstrated that inhibition of AKT enhanced the response of tumor cells to PARP inhibitor in ovarian cancer cell lines with high PARP1 protein expression level, corroborating the effect observed in PDX model study.

### Inhibition of AKT reduced PAR level and PARP1 protein level in vivo and in vitro

It has been reported that inhibition of PI3K/AKT/mTOR pathway induced HRD in the tumor cells [18]. YB-1, as a downstream molecule of PI3K/AKT/mTOR pathway, could stimulate PARP1 activity by interacting with DNA/RNA/PAR [31]. Thus, it is plausible that inhibition of AKT might affect PARP1 activity in the cells to increase anti-tumor activity of PARP inhibitor.

**Fig. 5:**
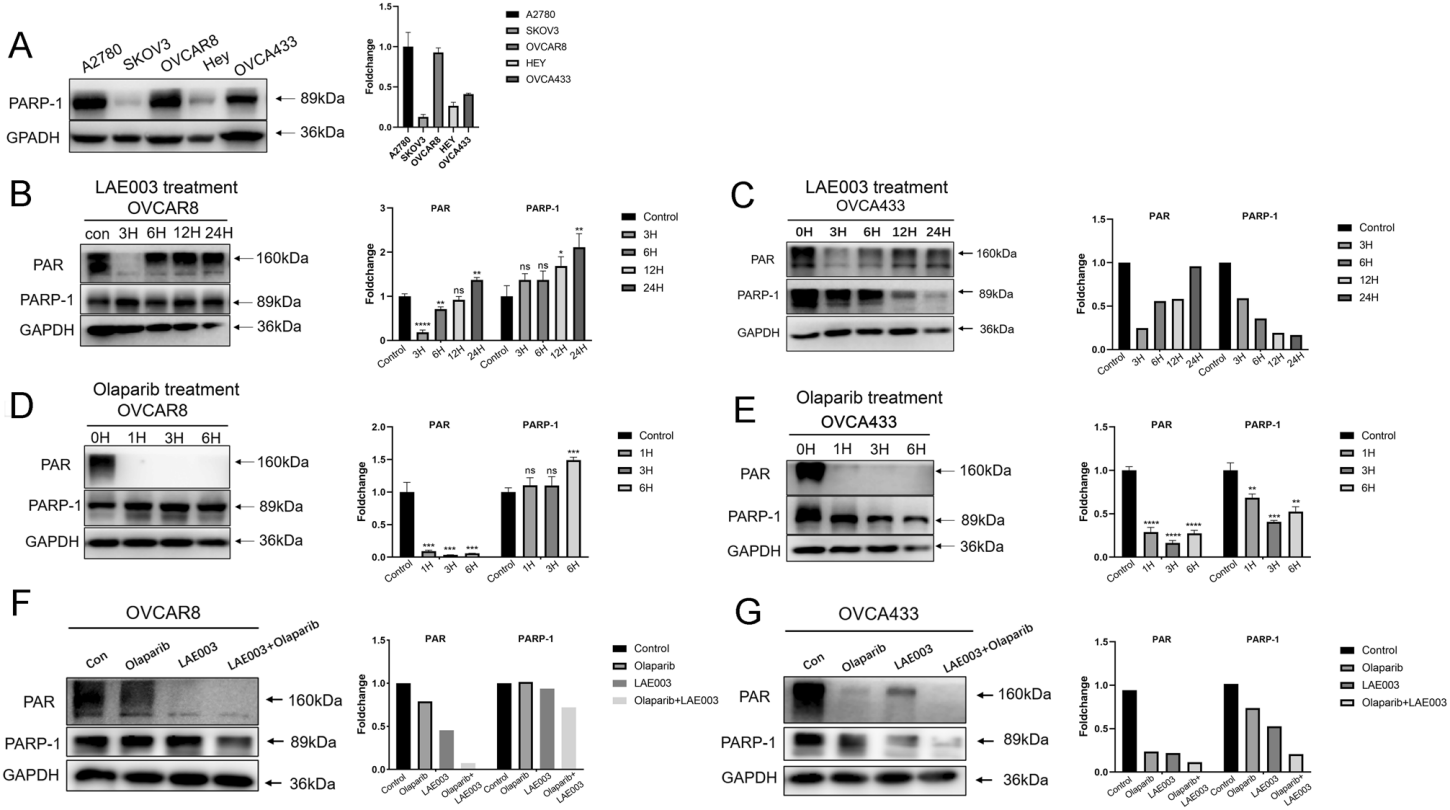
PAR and PARP1 protein levels were regulated by LAE003 or Olaparib treatment in ovarian cancer cell lines (**A**) Expression level of PARP1 protein in five ovarian cancer cells as assayed by Western blot (**B**) Western blot analysis of PARP1 and PAR levels in OVCAR8 cells after treated with LAE003 (15 µM) for various treatment duration. **C** Western blot analysis of PARP1 and PAR levels in OVCA433 cells after treated with LAE003 (15 µM) for various treatment duration. **D** Western blot analysis of PARP1 and PAR levels in OVCAR8 cells after treated with Olaparib (25 µM) for various treatment duration. **E** Western blot analysis of PARP1 and PAR levels in OVCA433 cells after treated with Olaparib (25 µM) for various treatment duration. **F** Western blot analysis of PARP1 and PAR levels in OVCAR8 cells after combination treatment of LAE003 (15 µM) and Olaparib (0.5 µM) for 3 h. **G** Western blot analysis of PARP1 and PAR levels in OVCA433 cells after combination treatment of LAE003 (15 µM) and Olaparib (0.5 µM) for 3 h. Densitometric analysis was performed for Western blot results. For the ones with two or more independent experiments, statistical analysis was conducted to determine the significance. ****P < 0.0001, ***P < 0.001, **P < 0.01, *P < 0.05 by Student’s *t* test

To test the hypothesis whether direct inhibition of AKT has any effect on PARP1 activity and protein level, two cell lines with high PARP1 level (OVCA433 and OVCAR8) were treated with 15 µM LAE003 or 25 µM Olaparib with various treatment duration (1–24 h). The cell lysates were collected to determine PAR and PARP1 levels using Western blot. As shown in Fig. 5D, E, Olaparib, by inhibiting PARP1 enzyme activity, decreased PAR level at all time points studied in both OVCA433 and OVCAR8 cells. PARP1 protein level was not changed significantly by Olaparib in OVCAR8 cells, while PARP1 protein level was reduced after the treatment of Olaparib in OVCA433 cells. Interestingly, AKT inhibitor LAE003 treatment also reduced the level of PAR in the cells. In OVCAR8 and OVCA433 cells, LAE003 had the most pronounced effect on reducing PAR level in the cells at 3-h treatment time point (Fig. 5B, C). At later treatment time points (6–24 h), PAR level in the cells gradually increased. Furthermore, PARP1 protein level was also reduced by LAE003 treatment in OVCA433 cells in a time dependent manner (Fig. 5C), while no change of PARP1 protein level was observed in OVCAR8 cells after LAE003 treatment (Fig. 5B).

To study whether combination treatment of LAE003 and Olaparib has additive effect on PARP enzyme activity or PARP1 protein level, OVCAR8 and OVCA433 cells were treated with 15 µM LAE003, 0.5 µM Olaparib or 15 µM LAE003/0.5 µM Olaparib combo for 3 h. To see the combination effect, a lower concentration of Olaparib (0.5 µM) was used in the study to partially reduce PAR level in the cells. As shown in Fig. 5F and G, LAE003 and Olaparib single treatment both partially reduced PAR level in the cells. There was more reduction of PAR level in the combination treatment group comparing single treatments, suggesting AKT inhibition and PARP inhibition additively reduced PAR level in the tumor cells.

**Fig. 6:**
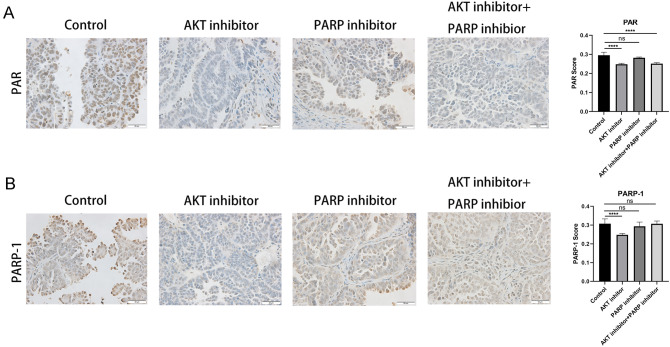
LAE003 treatment reduced PAR and PARP1 levels in the tumor tissues from PDX study. **A** Representative images of PAR IHC and quantification of PAR IHC score. **B** Representative images of PARP1 IHC and quantification of PARP1 IHC score. Data was presented as Means ± SD. (*n* = 5/group). ****P < 0.0001 comparing to vehicle group by Student’s *t* test

The effect of AKT inhibition on PAR and PARP1 protein levels were further supported by the PDX animal study. In the PDX study, after in-life phase monitoring tumor growth rate, tumor samples were collected to generate FFPE block. PAR and PARP1 IHC was performed on these samples. As shown in Fig. 6, a marked reduction of PAR and PARP1 protein levels was observed in LAE003 treatment group. A significant reduction of PAR level was also observed in LAE003/Olaparib treatment group. Olaparib single treatment did not affect PAR and PARP1 protein levels significantly. Taken together, these results suggest that inhibition of AKT may modulate the activity or the expression of PARP1 in the tumor cells. This could be another mechanism on how AKT inhibitor increases anti-tumor efficacy of PARP inhibitor in addition to enhancing HRD, although more studies are needed to elucidate the detailed signaling pathway changes after the use of AKT inhibitor.

## Discussions

The approval of PARP inhibitors has significantly changed the treatment landscape of EOC [32]. Although the use of PARP inhibitor as single agent treatment has been widely adopted, acquired resistance to PARP inhibition gradually emerges and becomes an area with high unmet medical need [14, 33]. Strategies to optimize the use of PARP inhibitor in a combination context, as well as to avoid and reverse the development of resistance have been investigated [17, 34]. AKT, as a central player of PI3K/AKT/mTOR pathway, becomes an attractive anti-tumor target and a potential combo partner for PARP inhibition [35–38]. Preliminary clinical activity has been observed in patients with advanced EOC treated with AKT inhibitor/PARP inhibitor combination [29, 39, 40]. In a phase I clinical trial, the combination of Capivasertib (an AKT inhibitor) and Olaparib (a PARP inhibitor) achieved clinical benefit (RECIST CR/PR or SD ≥ 4 months) in 11 out of 25 patients with advanced ovarian cancer [20]. Among these 25 EOC patients, five of them had prior PARP inhibitor use and resistance thereafter. One patient had a partial response to the combination. Although the number of patients evaluated was small, the clinical response observed in this trial is encouraging and supports the promise of the combination of AKT inhibitor and PARP inhibitor for ovarian cancer [20].

In our study, we further revealed the combination effect of PARP and AKT inhibitor in ovarian cancer using mini-PDX and PDX model. Different from the clinical trial of testing Capivasertib and Olaparib, we have particularly focused on recurrent EOC patients with platinum resistance and prior PARP inhibitor use, because these patients are the most difficult to treat and thus unmet medical need is huge. Utilizing Mini-PDX platform, we observed tumor cells from two out of five platinum-resistant EOC patients were sensitive to AKT inhibition (Table 2). A PDX model was subsequently generated using tumor samples from patient 3. In PDX model study, additive effect of AKT inhibitor treatment and PARP inhibitor treatment was observed, suggesting AKT inhibition could effectively increase the response of the tumor cells to PARP inhibitor. Although this PDX model was derived from a patient with confirmed resistance to PARP inhibitor, Olaparib single treatment also significantly slowed tumor growth in vivo. It is unclear why Olaparib had such effect on tumor growth. This may be due to the heterogenous nature of the tumor cells when doing biopsy collection. Due to the observed anti-tumor effect of Olaparib in PDX study, we could only conclude that AKT inhibition additively increased anti-tumor response of PARP inhibitor. Whether AKT inhibitor could re-sensitize the tumors to PARP inhibition should be further studied using other models with confirmed resistance to PARP inhibitor.

Based on the results of mini-PDX and PDX study using clinical samples, the combination effect of AKT inhibitor and PARP inhibitor was further studied in ovarian cancer cell lines. Additive effect on reducing cell viability was observed only in the cell lines with high PARP1 expression level (OVCA433, OVCAR8, A2780). In these three cell lines, cell apoptosis was further enhanced after the combo treatment. PARP1 expression is required for the efficacy of PARP inhibitor [41] and may drive anti-tumor activity of combination treatment with PARP inhibitor [30]. Based on in vitro study results, PARP1 protein level in ovarian cancer cells might be a potential marker to predict the combo effect of PARPi and AKTi. More studies are needed to confirm the finding.

Interestingly, inhibition of AKT appeared to reduce PARP1 protein level and PAR level in both PDX tumor tissue and ovarian cancer cell lines (OVCAR8 and OVCA433), although detailed mechanism is unclear. Expression level of PARP1 protein and activity of PARP1 enzyme has been reported to have an impact on the efficacy of PARP inhibitor. Previous mechanistic studies of PI3K/AKT/mTOR pathway inhibitor and PARP inhibitor combination mostly focused on the regulation of HR repair pathway. Our results suggested another mechanism that AKT inhibitor increased anti-tumor efficacy of PARP inhibitor through downregulating the activity and expression of PARP1. More studies should be carried out to understand molecular changes after AKT inhibitor in the cells and to evaluate whether there is any crosstalk between HR pathway and the pathway of PARP1 activity regulation.

The limitation of our study is the small sample size. Only five advanced EOC patient with platinum/PARP inhibitor resistance were studied. Studies with more patients are needed to further confirm the finding. Consistent with the clinical study of Capivasertib and Olaparib, our results suggest that the combination of AKT inhibitor and PARP inhibitor is likely to additively increase clinical response in recurrent EOC patients.

## Data Availability

The data generated during the present study are available from the corresponding author upon reasonable request.
